# Can Ensemble Deep Learning Identify People by Their Gait Using Data Collected from Multi-Modal Sensors in Their Insole?

**DOI:** 10.3390/s20144001

**Published:** 2020-07-18

**Authors:** Jucheol Moon, Nelson Hebert Minaya, Nhat Anh Le, Hee-Chan Park, Sang-Il Choi

**Affiliations:** 1 Department of Computer Engineering and Computer Science, California State University, Long Beach, CA 90840, USA; jucheol.moon@csulb.edu (J.M.); Nelson.Minaya@student.csulb.edu (N.H.M.); Nhat.Le01@student.csulb.edu (N.A.L.); 2 Department of Computer Science and Engineering, Dankook University, Yongin-si 16890, Korea; hchan11@naver.com

**Keywords:** gait analysis, user identification, deep learning, multi-modality, wearable sensors

## Abstract

Gait is a characteristic that has been utilized for identifying individuals. As human gait information is now able to be captured by several types of devices, many studies have proposed biometric identification methods using gait information. As research continues, the performance of this technology in terms of identification accuracy has been improved by gathering information from multi-modal sensors. However, in past studies, gait information was collected using ancillary devices while the identification accuracy was not high enough for biometric identification. In this study, we propose a deep learning-based biometric model to identify people by their gait information collected through a wearable device, namely an insole. The identification accuracy of the proposed model when utilizing multi-modal sensing is over 99%.

## 1. Introduction

Gait is a unique behavioral characteristic of an individual that can be used for identifying that person [1,2,3,4]. Studies [5,6,7] have demonstrated human’s ability to recognize individuals by their gait and have also confirmed that gait information is sufficient for discriminating between individuals. Several studies have looked into using gait information in the areas of disease diagnosis [8,9], and methods for user identification that utilize gait information have been proposed in recent years [10,11,12,13].

In general, user identification methods using gait information consist of two parts. The first part is collecting data representing gait information, and the second part is identifying the users by applying algorithms on the collected data. In this framework, we can categorize user identification methods by data collecting devices and user identifying algorithms. In detail, the gait information can be collected using vision sensors, pressure sensors, and Inertial Measurement Units (IMU), and the users are identified by applying Linear Discriminant Analysis (LDA), k-Nearest Neighbor (k-NN), Hidden Markov Model (HMM), Support Vector Machine (SVM), Convolutional Neural Network (CNN), or their combinations [14].

In this paper, we propose a framework to identify individuals from their gait information. We collect the gait information using sensors in insoles of shoes of participants, and we identify users using a combination of CNN and Recurrent Neural Network (RNN) [15]. We assume a special environment, in which all participants wear shoes with the insoles and their gait information is registered in the system. On this setting, our proposed framework can identify users from their gait information of one walking cycle with high accuracy.

The collected data consist of time series measured by pressure sensors, 3D-axis accelerometers, and 3D-axis gyroscopes installed in the insoles of shoes [16]. A walking cycle includes a stance phase and swing phase. The swing phase is the entire time a foot is in the air, therefore the pressure values during the swing phase should be zero for that foot [17]. Considering this characteristic of the walking cycle, we should be able to divide the original time series into a sequence of separate units, i.e., steps, to use the data more efficiently and effectively. However, the mounted pressure sensors in the insole frequently report non-zero values during the swing phase due to either interference between sensors or high temperatures [16]. To overcome potential errors from the pressure sensors, we determined the unit steps using Gaussian filtering [18].

To identify individuals from consecutive unit step data, we designed an ensemble network using CNN and RNN with multi-modal sensing data. The datasets were generated by selecting uni-, bi-, or tri-modal inputs from the pressure sensors, accelerometers, or gyroscopes. The CNN and RNN were then trained independently using identical training datasets. In the test phase, the softmax scores [19] of individuals are computed by taking averages of the softmax scores from the CNN and RNN [20]. Employing only single unit step, we achieved identification accuracy of around 99% using tri-modal sensing.

The contributions of this paper are as follows. (1) For the first time, the proposed model was able to identify people by utilizing deep learning algorithms to look at their gait information collected through a wearable device. (2) We proposed a novel method to detect the human gait cycle precisely from the raw gait information gathered. (3) We designed an ensemble model that uses CNN and RNN in cooperative and complementary manner. (4) The identification accuracy of the proposed model was maximized by utilizing multi-modal sensing.

This paper is organized as follows. Section 2 explains the data pre-processing procedure to transform the original gait information into standard format. Section 3 describes the design of the convolutional neural networks, recurrent neural network, and their ensemble model used for identification. Section 4 presents the experimental results. Section 5 includes discussion and future work. Section 6 concludes this paper.

### Related Work

Gait information has been analyzed to diagnose mental and physical diseases. Changes of gait pattern may be indexes of intellectual disability [21], dementia [22], depression [23], and deformation of the joint diseases [24]. Gait information can reflect an older adults’ mental health; in particular, when their mental health is negative, their gait becomes more asymmetrical [25]. In addition, gait analysis can also be used for age and gender estimation [26]. These insights affect a variety of research areas such as healthcare [27], biomedical [28], and sports [29]. Studies [8,9] have demonstrated that patients who have Parkinson’s disease can be recognized by analyzing their gait information. As an extension of such studies, types of gait, i.e., walking, running, climbing, and descending, have been successfully classified using gait information, which was collected from wearable sensors [30]. In other research, Dynamic Bayesian Networks could classify different types of gait, i.e., normal, left limp, and right limp, using data recorded by Microsoft Kinect V2 system [31].

Gait analysis as a means of identifying individuals, i.e., biometrics, began by utilizing vision sensors [32]; these approaches have since been further studied and evolved [33,34,35,36]. However, vision-based gait analysis requires strict conditions during sensing, for example the video sequences must include only the individual to be identified, and its identification accuracy is not high enough to use it as biometric tool by itself. Furthermore, the accuracy depends on the perspectives and orientation of the sensing devices. To overcome these shortcomings, pressures sensors and IMU are widely used to collect gait information. In general, IMUs consist of accelerometers, gyroscopes, and magnetometers. In [37], gait cycles of a user were monitored by a sensor network that used a pair of wireless IMU sensors. In [10], gait information was collected from five IMUs placed on the chest, lower back, right wrist, knee, and ankle of the participants. To identify individuals, a CNN-based predictive model was used [38]. This network took the measured time-domain data and their transformed frequency domain data together as inputs.

Recently, pressure sensors and IMUs have been installed in wearable devices such as smartphones, fitness trackers, or in the insoles of shoes [11]. In [12], IMUs installed in smartphones were utilized to gather gait information. Data were collected while participants carried their phones in their front trouser pockets, then a mixture model of a CNN and a SVM [39] was used to identify individuals. In [13], gait information was gauged by using pressure sensors and accelerometers in the insoles of shoes. The collected data were analyzed using null space linear discriminant analysis [40] to identify individuals. However, these methods use a few different types of sensors placed on various parts of the body, require a long period of time for gathering data, or need to be improved in terms of identification accuracy. In addition, collecting data using wearable devices can be more invasive than using vision-based devices.

## 2. Gait Information

Gait information is collected as time series vectors and represent multiple consecutive unit steps. Instead of using the data as they are, we divided them into fixed size fragments to improve identification accuracy and reduce computational complexity.

### 2.1. Data Source

**Figure 1:**
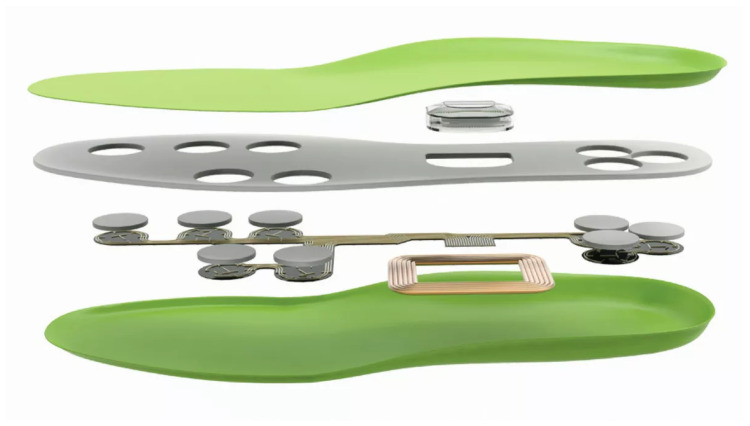
The architecture of the FootLogger insole.

We used a commercial insole, FootLogger, to collect gait information. As shown in Figure 1, FootLogger includes eight pressure sensors, a 3D-axis accelerometer, and a 3D-axis gyroscope for each foot. The sampling rate is 100 Hz for each device. The pressure sensor categorizes the intensity of pressure to one of three levels: 0, 1, or 2. The accelerometer and gyroscope measure acceleration and rotation, respectively; both are recorded in 3D space as integers between −32,768 and 32,768.

We denote (uni-variate) time series and multivariate time series by x(t) and x(t), respectively. Different sensing modality is expressed by using superscript letters, i.e., $x^{p}\left(t\right)$ for pressure, $x^{a}\left(t\right)$ for acceleration, and $x^{r}\left(t\right)$ for rotation. Different participant identification is expressed by using subscript numbers, i.e., $x_{i}\left(t\right)$ for id=i.

### 2.2. Data Pre-Processing

By transforming the collected data into a fixed standard format, we can expect better performance in terms of identification accuracy [13,16]. To convert the data into the standard format, we propose a novel data standardization method using Gaussian filtering.

#### 2.2.1. Walking Cycle Detection

**Figure 2:**
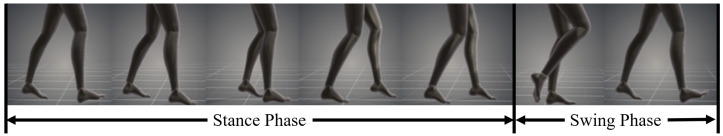
Typical human walking cycle.

As shown in Figure 2, a typical human walking cycle can be broken down into two phases: the stance phase and the swing phase [17]. The stance phase is the whole time that a foot is on the ground, and the swing phase is the entire time a foot is in the air. Since the pressure sensors in the insole measure pressure between the foot and the ground, the pressure values collected by all eight pressure sensors should be zeros during swing phases. Using this information, we should be able to divide the original time series data into consecutive unit steps. Intuitively, we might set the beginning of a unit step to the time when the mean of the eight pressure values changes to zero from a non-zero value. However, this approach highlights a latent problem of the system where non-zero pressure values are frequently recorded during swing phases. This phenomenon can be caused by interference between sensors or high temperatures in shoes [16].

**Figure 3:**
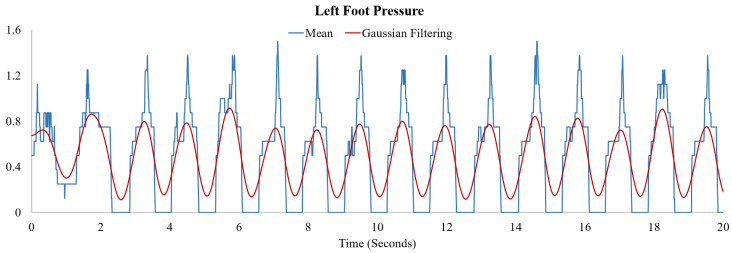
Mean of eight pressure values (blue lines) and convolution of the mean and Gaussian function with standard deviation σ=0.2 s (red lines).

To determine the unit steps more consistently and precisely, we perform a convolution operation with the mean of the eight pressure values and the Gaussian function. Let $x^{p}\left(t\right)$ be the mean of eight pressure values and $y\left(t\right)=\frac{1}{\sqrt{2\pi }\sigma }e^{-\frac{t^{2}}{2\sigma^{2}}}$ where σ=0.2 s. The convolution operation is defined as $z\left(t\right)=\left(x^{p}\left(t\right)*y\right)\left(t\right)=\int_{0}^{t}x^{p}\left(\tau \right)y\left(t-\tau \right)d\tau$. In Figure 3, examples of x(t) and z(t) are denoted by blue lines and red lines, respectively.

#### 2.2.2. Standard Format

We define the unit step formally. For each foot, we consider an ordered list $\left[t_{0},t_{1},\ldots ,t_{i},\ldots \right]$ where $t_{i}<t_{i+1}$ for all time *t* such that $\frac{d}{dt}z\left(t\right)=0$ and $\frac{d^{2}}{dt^{2}}z\left(t\right)>0$. We define unit step as time series $s_{i}\left(t\right)=x\left(t+t_{i}\right)$ where $0\leq t<t_{i+1}-t_{i}$. For discrete variables, we define $len\left(s_{i}\left(t\right)\right)=\left\lfloor \left(t_{i+1}-t_{i}\right)\times 100\right\rfloor$ since the sampling rate of the insole is 100 Hz, and the standard length is defined by $d=\min_{i}len\left(s_{i}\left(t\right)\right)$ where $s_{i}\left(t\right)$ are unit steps of both feet of all participants. Then, we resize all unit steps into the standard length *d* using spline interpolation [41], and the unit steps of identical modalities for both feet are concatenated. In summary, *i*th unit step for sensing modality *m* is denoted by $s_{i}^{m}\left(t\right)$ where m∈{p,a,r}, and their dimensions are $|s_{i}^{p}\left(t\right)|=d\times \left(8\cdot 2\right)$ and $|s_{i}^{a}\left(t\right)|=|s_{i}^{r}\left(t\right)|=d\times \left(3\cdot 2\right)$. We set d=87 in the experiments.

**Figure 4:**
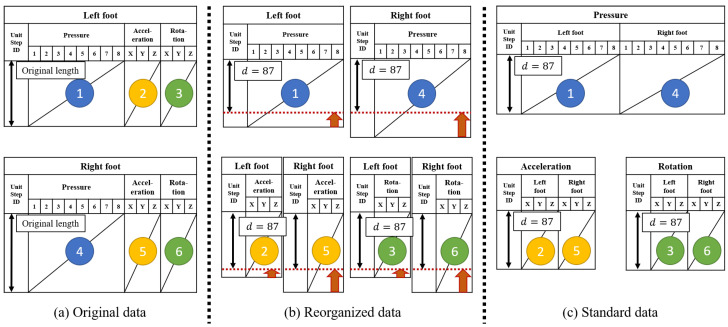
The procedure of data standardization. (**a**) Original format. (**b**) The length of the format is fixed as d=87. (**c**) The same sensing modalities of both feet are concatenated in the standard format.

To show data standardization intuitively, the procedure is illustrated in Figure 4. the original time series for both feet was converted into the standard format by the following procedure: (a) The original time series was divided into the unit fragments using the start time and end time of the unit steps of the both feet independently. (b) All unit fragments were resized to the standard unit step interval *d* using spline interpolation [41]. They were then separated into three sensing modes: pressure, acceleration, and rotation. (c) For each sensing mode, the resized fragments for both feet were joined together.

In addition, we generated reference datasets by combining *k* (for 1<k≤4) consecutive unit steps into one sample. With using the reference datasets, we show the effect of the amount of information in a unit step to the classification accuracy. The amount of information is proportional to the *k*-value. In detail, we re-labeled the series of unit steps $s_{i}\left(t\right),\cdots ,s_{i+k-1}\left(t\right)$ to a new label $s_{j}\left(t\right)$ for all i∈{0,k,2k,…} where $j=\lfloor \frac{i}{k}\rfloor$. Therefore, the dimensions of the standard datasets can be expressed as (k·d)×(w·2) where w=8 for pressure and w=3 for acceleration and rotation.

## 3. Network Design

The standard datasets include time series which are measured using tri-modal sensing. We assumed that the eight pressure values are correlated since they gauged pressure between different positions of a single foot and the ground during the same gait cycle in the insole. Similarly, we supposed that the acceleration values in three-dimensional space are correlated, and the rotation values in three-dimensional space are correlated as well. By considering these characteristics, we designed a predictive network model utilizing CNN and RNN. The input of the proposed network model is the unit steps of pressure $s_{i}^{p}\left(t\right)$, acceleration $s_{i}^{a}\left(t\right)$, and rotation $s_{i}^{r}\left(t\right)$ in the standard format, and the output of the model is the vector of softmax probabilities **u**:

(1) $$M\left(s_{i}^{p}\left(t\right),s_{i}^{a}\left(t\right),s_{i}^{r}\left(t\right)\right)=u\;\left(\mathrm{for}\;\mathrm{tri}-\mathrm{model}\;\mathrm{sensing}\right)$$

**Figure 5:**
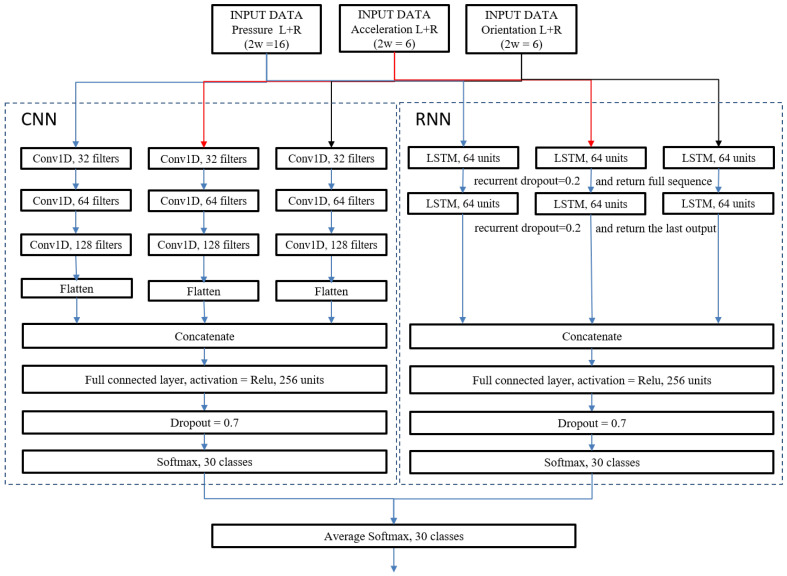
The architecture of the proposed network model.

We used the notation $M_{cnn}$ when only the CNN is activated, $M_{rnn}$ when only the RNN is activated, and $M_{ens}$ when both CNN and RNN are activated. In addition, the proposed network model is designed to also be applicable to uni-modal and bi-modal sensing. We used notations $M\left(s_{i}^{p}\left(t\right)\right)$, $M\left(s_{i}^{a}\left(t\right)\right)$, $M\left(s_{i}^{r}\left(t\right)\right)$ for the uni-modal sensing model and used $M\left(s_{i}^{p}\left(t\right),s_{i}^{a}\left(t\right)\right)$, $M\left(s_{i}^{p}\left(t\right),s_{i}^{r}\left(t\right)\right)$, $M\left(s_{i}^{a}\left(t\right),s_{i}^{r}\left(t\right)\right)$ for the bi-modal sensing model. A conceptual diagram of the network model is depicted in Figure 5.

### 3.1. Convolutional Neural Network

The proposed CNN consists of independent but identical layers for each sensing mode. The network includes three consecutive one-dimensional (1D) convolution layers. The first convolution layer contains 32 filters whose sizes are 20×(w·2), the second layer contains 64 filters whose sizes are 20×32, and the third layer contains 128 filters whose sizes are 20×64. For the first layer, the width of the filters equals the width of the standard input format (w·2). For the second and third layers, the width of the filters equals the number of the filters in the previous convolutional layer. The convolution operation between a filter and the input produces a single scalar value, and the series of scalar values are concatenated to give a feature vector. The filtering stride for all convolutional layers was set to 1, while the padding sizes were determined to keep the output to the same height as the input. Therefore, the dimensions of the feature maps after each of the three convolutional layers are 87×32, 87×64, and 87×128. After the third convolutional layer, the feature map is flattened to generate a feature vector whose size is (87, 128). For bi-modal and tri-modal sensing, the feature vectors from different sensing modes are concatenated to one vector which becomes the input for the fully connected layer. To avoid the vanishing gradient phenomenon, we used the Rectifier Linear Unit (ReLU) as the activation function [42].

### 3.2. Recurrent Neural Network

Similar to the CNN, the proposed RNN consists of identical layers for each sensing mode. The network model includes two consecutive Long Short-Term Memory (LSTM) [43] layers. Unlike other RNN models, LSTM is applicable to time series data over a long period of time by utilizing internal memory units which are designed to overcome the ‘error back-flow problem’ [43]. In the proposed network, each LSTM layer contains 64 memory units whose forget, input, and output gates are activated using the hard sigmoid function. We set the recurrent dropout ratio as 0.2 to prevent the over-fitting problem [44].

The dimension of the input for the first LSTM layer is (k·d)×(w·2). At a given row of the input data, the first LSTM layer is designed to produce a single scalar value per memory unit, the series of scalar values are then concatenated to give an output vector. Hence, the dimension of the output from the first LSTM layer is (k·d)×64, which is the input for the second LSTM layer. Unlike the first LSTM layer, the second LSTM layer returns only the last scalar value in the output vector per memory unit. Therefore, the dimension of the output for the second LSTM layer is just 64. For bi-modal and tri-modal sensing, the output vectors from different sensing modes are concatenated as one vector which becomes the input for the fully connected layer.

### 3.3. Fully Connected Network

The fully connected network includes a fully connected layer, a dropout layer, and a softmax layer. The fully connected layer contains 256 nodes, and the activation function of the nodes is ReLU. Individual nodes are kept with a probability 0.7, so that a reduced layer is left, which is denoted by the dropout layer in Figure 5. This ‘dropout’ procedure is used to alleviate the over-fitting problem and improve regularization performance [44]. The softmax layer contains *n* nodes where *n* is the number of user identifiers, in this way a user identifier can be selected by computing the normalized probabilities using the softmax function.

### 3.4. Averaging Ensemble Model

The proposed network model utilizes CNN and RNN independently to select a user identifier. To aggregate the predictions from the CNN and RNN and give one final prediction, we suggest an averaging ensemble model. Since both CNN and RNN have the same softmax layer at the end, we can compute the averaging probabilities of CNN and RNN by

(2) $$M_{ens}=\frac{1}{2}\left(M_{cnn}+M_{rnn}\right)$$

We do not include the results here, but we also implemented a two-dimensional linear SVM ensemble model. Despite its computational complexity being significantly higher than the averaging ensemble model, the prediction accuracy of the linear SVM ensemble model is not better than the averaging ensemble model.

## 4. Experimental Results

We show the performance of our proposed method using empirical datasets. We compared the identification accuracies under distinct sensing modalities (single, double, and triple) and different network designs (CNN, RNN, and Ensemble).

### 4.1. Datasets and Evaluation Method

We collected gait information from 30 adults aged 20–30 years old. The data were measured using the insole while the participants walked for about 3 min. During the 3 min of walking, the data collected included approximately 160 unit steps on average, and the entire dataset encompassed 4750 unit steps in total. In the experiments, we determined d=87; hence, the dimensions of the datasets for pressure, acceleration, and rotation are (k·87)×(8·2), (k·87)×(3·2), and (k·87)×(3·2), respectively, for 1≤k≤4.

**Figure 6:**
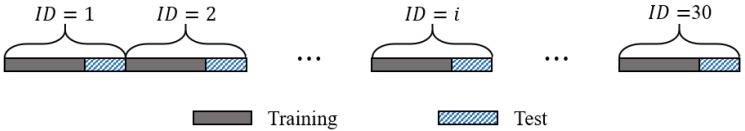
Illustration of splitting data into training and test datasets.

As shown in Figure 6, we generated the training and testing datasets independently for three types of Monte Carlo Cross-Validation (MCCV) [45] methods, which are MCCV (30%), Sub-MCCV (50%), and MCCV (50%). Sub-MCCV sets were created to see the performance of the system with the limited number of samples in the training dataset. MCCV utilized 100% of samples in either training or test dataset, but Sub-MCCV used only about 84% of samples in total. The first dataset was generated by selecting 30% of the overall sample as the testing data and the remaining ones as the training data, and we denote this set as MCCV (30%). The second dataset was generated from a subset of the overall samples by selecting the same number of samples for the testing and training data, and we denote this set as Sub-MCCV (50%). The third dataset was generated by selecting 50% of overall samples as the testing data and the remaining ones as the training data, and we denote this set as MCCV (50%). The number of samples for the different *k*-values and validation methods are summarized in Table 1. We repeated generating the three types of datasets 20 times by following the procedure explained above. For each dataset, our proposed networks were trained and tested independently, and then the averaged identification accuracies were summarized for each type of the MCCV methods.

### 4.2. Latent Space and Training Time

**Figure 7:**
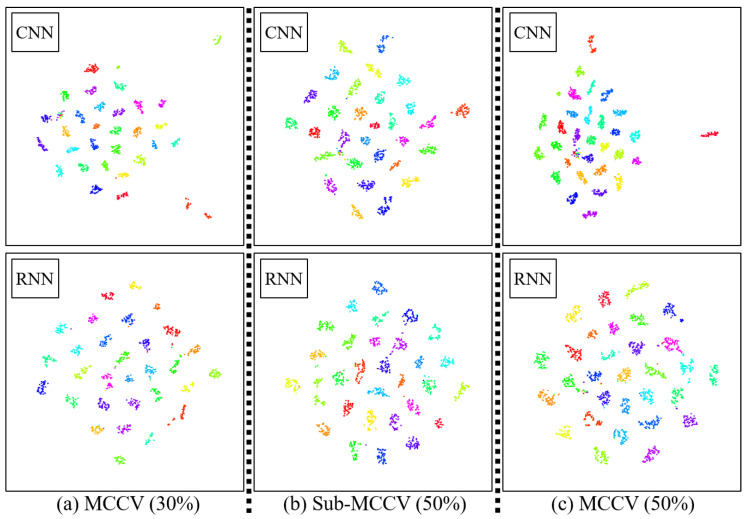
t-SNE plots of the output of the fully connected layer with 256 units in the CNN and RNN architectures. (**a**) MCCV (30%), (**b**) Sub-MCCV (50%), (**c**) MCCV (50%).

We show the t-distributed stochastic neighbor embedding (t-SNE) plots of feature vectors of unit steps in Figure 7. t-SNE performs nonlinear dimensionality reduction [46]. The feature vector is the output of the fully connected layer with 256 units in the CNN and RNN architectures in Figure 5. In Figure 7, each color represents a participant, and the dots with the same color correspond to the unit steps of the participant. In general, all unit steps of each participant are clearly grouped together, and unit steps of every participant are clearly clustered.

All experiments were performed on a workstation with Intel Core i9-9820X Skylake X 10-Core 3.3 GHz CPU (Intel, Santa Clara, CA, USA), ASUS ROG GeForce RTX 2080 Ti GPU (Asus, Taipei, Taiwan), and Samsung 64GB DDR4 PC4-21300 2666MHZ RAM (Samsung, Suwon, Korea). For k=1 and the tri-modal sensing, the average training time of the ensemble model was 28.48 min.

### 4.3. Identification Accuracy

Using the generated dataset, we evaluated the performance of our proposed framework considering three scenarios, namely single, double, and triple sensing modalities, from pressure, acceleration, and rotation data. Using a given training dataset generated by one of the three different MCCV methods, we trained the CNN and RNN networks independently for the distinct *k*-values of 1–4. The predictive results of the ensemble network were determined by averaging the softmax scores of the pre-trained CNN and RNN networks.

#### 4.3.1. Tri-Modal Sensing

**Figure 8:**
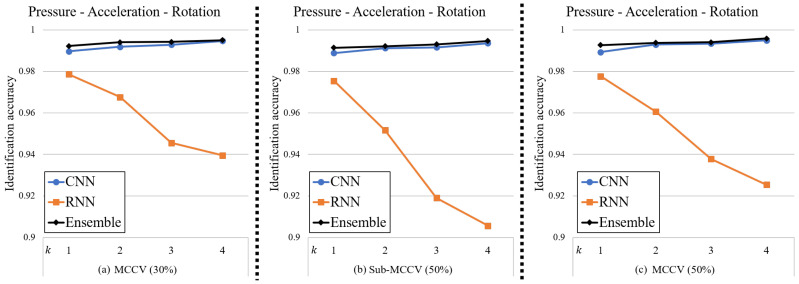
Identification accuracies of the proposed method using tri-modal sensing for different *k*-values. (**a**) MCCV (30%), (**b**) Sub-MCCV (50%), (**c**) MCCV (50%).

The first scenario considered was that we were able to utilize all modalities of pressure, acceleration, and rotation data. Figure 8 depicts the identification accuracies for the CNN, RNN, and ensemble networks using the tri-modal sensing. Detailed information about the identification accuracies is summarized in Table 2.

For all types of dataset, the CNN network is overwhelmingly more accurate than the RNN network for identification. Interestingly, the identification accuracy of the RNN network declines with increasing *k*-value while the accuracy of the CNN network increases. These trends mean, for larger *k*-values, the identification accuracy of the CNN network is particularly outstanding compared to the RNN network. However, the identification accuracy of the ensemble network is slightly higher than the CNN network alone, even when the identification accuracy of RNN is relatively low.

#### 4.3.2. Bi-Modal Sensing

**Figure 9:**
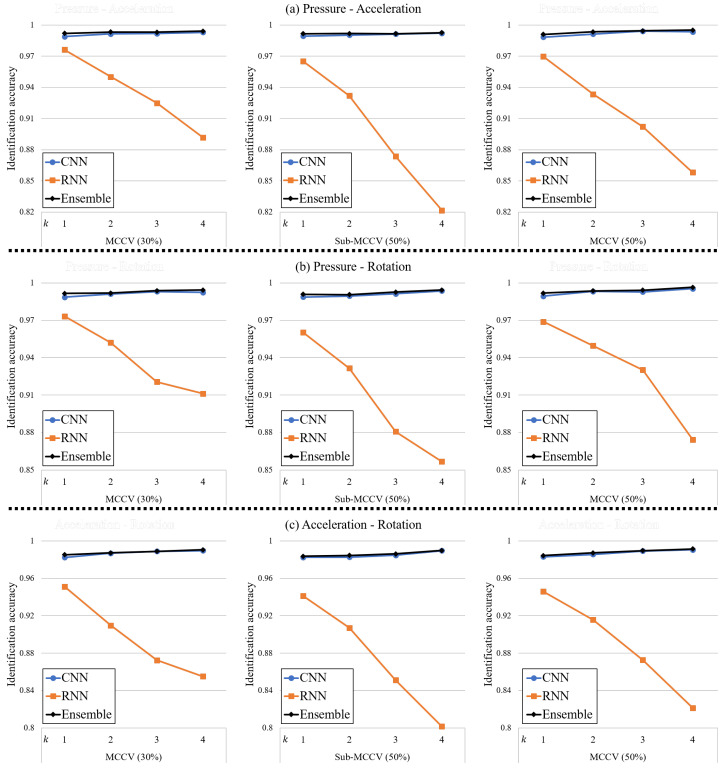
Identification accuracies of the proposed method using bi-modal sensing for different *k*-values. (**a**) Pressure - Acceleration, (**b**) Pressure - Rotation, (**c**) Acceleration - Rotation.

The second scenario considered was that we were able to utilize a combination of two modalities out of pressure, acceleration, and rotation data. Figure 9 depicts the identification accuracies for the CNN, RNN, and ensemble networks using bi-modal sensing. For the sake of brevity, we summarize the identification accuracies for only k=1 in Table 3.

Considering the results of tri-modal sensing, it is difficult to argue that the identification accuracy of our proposed method using bi-modal sensing is lower than for tri-modal sensing. In addition, we could not figure out why the identification accuracy of the model using certain combinations of sensing modalities outperformed other combinations because the accuracy of all combinations is excellent.

#### 4.3.3. Uni-Modal Sensing

**Figure 10:**
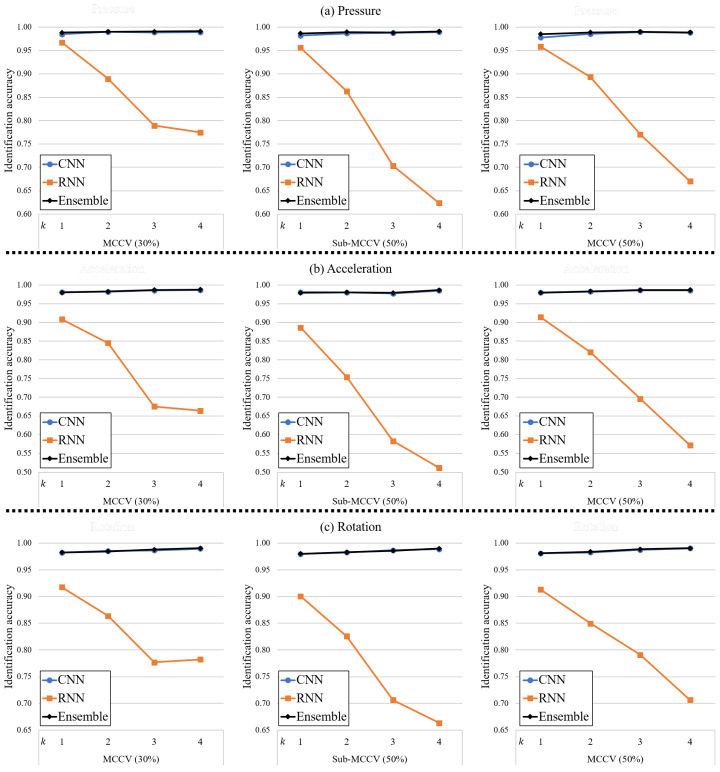
Identification accuracies of the proposed method using uni-modal sensing for different *k*-values. (**a**) Pressure, (**b**) Acceleration, (**c**) Rotation.

The third scenario considered was that we were able to utilize one modality from pressure, acceleration, and rotation data. Figure 10 depicts the identification accuracies for the CNN, RNN, and ensemble networks using uni-modal sensing. Similar to the bi-modal sensing results, we summarize the identification accuracies for only k=1 in Table 3.

Since all of the identification accuracies for the network using bi-modal and tri-modal sensing with k=1 are higher than 99%, we can say that the identification performance of the model using uni-modal sensing is slightly lower than for bi-modal or tri-modal sensing. However, we would emphasize that the identification accuracies of all the models using uni-modal sensing are excellent.

## 5. Discussion and Future Work

**Figure 11:**
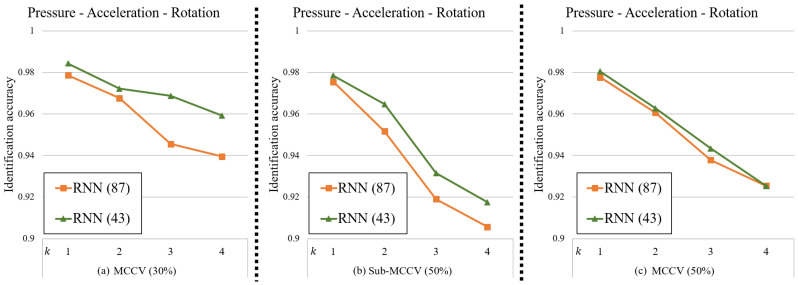
Identification accuracies of the RNN with two hyper-parameters, d=87 and d=43, using tri-modal sensing for different *k*-values. (**a**) MCCV (30%), (**b**) Sub-MCCV (50%), (**c**) MCCV (50%).

From the experimental results, we showed that the identification accuracies of the CNN generally improved as the number of consecutive unit steps, *k*, increased. On the other hand, the identification accuracies of the RNN decreased as the value of *k* increased. From the above results, we hypothesize that the identification accuracies of the RNN would increase as the value of *d* decreases in general. To verify this, we generated a new dataset with a standard format length value d=43 from the dataset used in the above experiment by spline interpolation. Using this new dataset, an experiment of tri-modal sensing using only the RNN model was repeated and the results are summarized in Figure 11.

As expected, we were able to verify that the accuracies of the RNN with d=43 is higher than that with d=87 for all *k*-values. Therefore, in future research, we will look to optimize the hyper-parameter *d* value of the RNN differently from the *d* value of the CNN to improve the overall accuracies of the ensemble model. In addition, we plan to conduct research that extends our framework to user authentication application.

## 6. Conclusions

This paper presents a new method to identify people by their gait information collected using multi-modal sensors in the insoles of their shoes. The insole equipped three types of sensors, which measure pressure, acceleration, and rotation. Using the pressure data, our proposed method successfully detected the human gait cycle precisely even though the original gait information includes noise from sensors. To identify individuals using the data of their single gait cycles, we propose an ensemble model based on CNN and RNN, which employs the different sensing data either separately or together. Considering identification accuracies, the ensemble model showed the highest performance compared to CNN or RNN alone. In terms of the sensor types, the model with tri-modal sensing showed highest accuracies, and the model with bi-modal or uni-modal sensing showed slightly lower accuracies. Since these insoles can be installed in any type of shoes, our framework could be used for a wide variety of applications including user authentication and verification.

## Figures and Tables

**Table 1:** The total number of samples for different *k*-values. MCCV stands for Monte Carlo cross-validation.

*k*	# of Samples	MCCV (30%)		Sub-MCCV (50%)		MCCV (50%)	
		Train	Test	Train	Test	Train	Test
1	4750	3325	1425	2000	2000	2375	2375
2	2368	1658	710	1000	1000	1184	1184
3	1570	1099	471	600	600	785	785
4	1176	823	353	500	500	588	588

**Table 2:** Identification accuracies of the proposed method using tri-modal sensing (pressure, acceleration, and rotation) for different *k*-values.

Validation	*k*	$M_{\mathrm{cnn}}$	$M_{\mathrm{rnn}}$	$M_{\mathrm{ens}}$
MCCV (30%)	1	0.9896	0.9786	0.9922
	2	0.9918	0.9676	0.9940
	3	0.9928	0.9456	0.9942
	4	0.9947	0.9395	0.9950
Sub-MCCV (50%)	1	0.9888	0.9754	0.9914
	2	0.9912	0.9516	0.9921
	3	0.9915	0.9190	0.9930
	4	0.9935	0.9056	0.9946
MCCV (50%)	1	0.9892	0.9776	0.9926
	2	0.9929	0.9606	0.9937
	3	0.9934	0.9378	0.9940
	4	0.9949	0.9253	0.9958

**Table 3:** Identification accuracies of the proposed method using bi-modal sensing (combinations of two of pressure, acceleration, and rotation) for *k* = 1.

Validation	Sensing	$M_{\mathrm{cnn}}$	$M_{\mathrm{rnn}}$	$M_{\mathrm{ens}}$
MCCV (30%)	$x_{p}\left(t\right),x_{a}\left(t\right)$	0.9888	0.9762	0.9919
	$x_{a}\left(t\right),x_{r}\left(t\right)$	0.9823	0.9512	0.9853
	$x_{r}\left(t\right),x_{p}\left(t\right)$	0.9886	0.9732	0.9916
Sub-MCCV (50%)	$x_{p}\left(t\right),x_{a}\left(t\right)$	0.9893	0.9651	0.9917
	$x_{a}\left(t\right),x_{r}\left(t\right)$	0.9825	0.9412	0.9836
	$x_{r}\left(t\right),x_{p}\left(t\right)$	0.9887	0.9603	0.9908
MCCV (50%)	$x_{p}\left(t\right),x_{a}\left(t\right)$	0.9884	0.9697	0.9910
	$x_{a}\left(t\right),x_{r}\left(t\right)$	0.9831	0.9459	0.9844
	$x_{r}\left(t\right),x_{p}\left(t\right)$	0.9893	0.9688	0.9918
MCCV (30%)	$x_{p}\left(t\right)$	0.9847	0.9673	0.9885
	$x_{a}\left(t\right)$	0.9808	0.9086	0.9798
	$x_{r}\left(t\right)$	0.9821	0.9173	0.9824
Sub-MCCV (50%)	$x_{p}\left(t\right)$	0.9818	0.9559	0.9867
	$x_{a}\left(t\right)$	0.9808	0.8860	0.9789
	$x_{r}\left(t\right)$	0.9796	0.9004	0.9800
MCCV (50%)	$x_{p}\left(t\right)$	0.9777	0.9580	0.9851
	$x_{a}\left(t\right)$	0.9801	0.9139	0.9796
	$x_{r}\left(t\right)$	0.9808	0.9130	0.9808
